# Probabilistic sequence learning in mild cognitive impairment

**DOI:** 10.3389/fnhum.2013.00318

**Published:** 2013-07-01

**Authors:** Dezso Nemeth, Karolina Janacsek, Katalin Király, Zsuzsa Londe, Kornél Németh, Kata Fazekas, Ilona Ádám, Király Elemérné, Attila Csányi

**Affiliations:** ^1^Department of Clinical Psychology and Addiction, Institute of Psychology, Eötvös Loránd UniversityBudapest, Hungary; ^2^Institute of Psychology, University of SzegedSzeged, Hungary; ^3^American Language Institute, University of South-CaliforniaLos Angeles, USA; ^4^Department of Cognitive Sciences, Budapest University of Technology and EconomicsBudapest, Hungary; ^5^Aladár Petz County Research HospitalGyor, Hungary

**Keywords:** mild cognitive impairment, offline learning, statistical learning, implicit learning, skill learning, consolidation, automaticity

## Abstract

Mild Cognitive Impairment (MCI) causes slight but noticeable disruption in cognitive systems, primarily executive and memory functions. However, it is not clear if the development of sequence learning is affected by an impaired cognitive system and, if so, how. The goal of our study was to investigate the development of probabilistic sequence learning, from the initial acquisition to consolidation, in MCI and healthy elderly control groups. We used the Alternating Serial Reaction Time task (ASRT) to measure probabilistic sequence learning. Individuals with MCI showed weaker learning performance than the healthy elderly group. However, using the reaction times only from the second half of each learning block—after the reactivation phase—we found intact learning in MCI. Based on the assumption that the first part of each learning block is related to reactivation/recall processes, we suggest that these processes are affected in MCI. The 24-h offline period showed no effect on sequence-specific learning in either group but did on general skill learning: the healthy elderly group showed offline improvement in general reaction times while individuals with MCI did not. Our findings deepen our understanding regarding the underlying mechanisms and time course of sequence acquisition and consolidation.

## Introduction

Mild cognitive impairment (MCI) is a transition stage between normal age-related cognitive decline and the more serious symptoms of dementia caused by, for example, Alzheimer's disease. According to the American College of Physicians, MCI affects about 20% of the population over 70 years of age. Many who develop MCI eventually develop Alzheimer's disease, although some will remain stable or might even return to normal (Roberts et al., 2008). Of those with MCI, 12–15% will develop the signs of dementia within a year and about 50% will progress to dementia within 5 years (Gauthier et al., 2006). The characteristic symptoms of MCI are impaired memory functions during learning or recall, impaired attention and information processing evidenced by the speed with which these functions are executed, flawed executive functions, and perceptual motor-skill and language-expression disturbances (e.g., word finding). MCI is diagnosed if at least two of these symptoms are present for at least 2 weeks (Tariska et al., 1990; Petersen et al., 1999; Grundman et al., 2004; Portet et al., 2006). MCI produces greater than age appropriate memory impairment but in all other aspects the individual functions well. Most often, learning skills and the ability to recall new information are affected to the highest extent. Brain imaging research shows dysfunction in the medial temporal lobe (MTL), including the hippocampal formation in MCI (Jack et al., 1997; Dickerson and Sperling, 2008; Nickl-Jockschat et al., 2012; Szamosi et al., 2013) but other areas might also be affected (Rombouts et al., 2009). Memory tests have established that certain forms of explicit memory and learning, such as delayed recall and list learning, decline in MCI (Petersen et al., 1999; Grundman et al., 2004; Leube et al., 2008). However, the question of how implicit learning is affected by MCI has received less attention (Nagy et al., 2007; Negash et al., 2007b). Properties of implicit learning and its consolidation could be useful in the dissociation of MCI from healthy age-related changes and also could contribute to a better understanding of the formation and consolidation of sequence acquisition, specifically the role of the MTL and hippocampus in these processes.

Explicit or declarative memory is accessible to conscious recollection, including facts and episodes (for example remembering events explicitly). It is defined by voluntary mechanisms which rely more on attentional resources. Non-declarative memory relies more on automatic, non-conscious/implicit processes including habituation, conditioning, motor and perceptual skills (for example playing piano). According to Squire and his colleagues, explicit or declarative memory can be linked to the brain's medial-temporal area, while the implicit or non-declarative processes fall outside these areas (Squire and Zola, 1996; Squire, 1998). Nevertheless, others showed that areas in the MTL including hippocampus also play a role in implicit learning (Chun and Phelps, 1999; Albouy et al., 2008; for critics, see Manns and Squire, 2001; Poldrack and Rodriguez, 2003).

The focus in our study is primarily on implicit sequence learning which underlies the acquisition of not only motor but also cognitive and social skills (Lieberman, 2000; Nemeth et al., 2011; Romano Bergstrom et al., 2012). Most models of sequence learning (Hikosaka et al., 1999, 2002; Doyon et al., 2009a) emphasize the role of the frontal-striatal-cerebellar system, while the role of the MTL and related structures (e.g., hippocampus) remains inconclusive (Schendan et al., 2003; Albouy et al., 2008; Simon et al., 2012). Negash et al. (2007b) have conducted the first and only research to address this topic so far, in which they investigated the effect of MCI on implicit learning. They used two implicit learning paradigms: the Serial Reaction Time (SRT; Nissen and Bullemer, 1987) to measure sequence learning, and the Contextual Cueing Task (Chun and Jiang, 1998) to measure visuospatial configuration learning. Despite the similarity in implicitness of these tasks, they call on two different neural systems; previous studies showed greater involvement of MTL in the Contextual Cueing (Chun and Jiang, 1998; Manns and Squire, 2001) compared to the SRT task, which is primarily mediated by the previously mentioned frontal-striatal-cerebellar system (Curran, 1998; Honda et al., 1998; Gomez-Beldarrain et al., 1999; Willingham et al., 2002). Negash et al.'s results revealed that individuals with MCI, although generally slower, showed similar sequence learning to the controls; however, learning was impaired in the Contextual Cueing task. These findings implicate that the MTL system, including the hippocampal formation is involved in MCI, while the frontal-striatal-cerebellar system is involved to a lesser extent (Negash et al., 2007b).

While Negash et al. (2007b) used a deterministic 8-element sequence, we take the task one step further. Here we use a modified version of the SRT task, the Alternating Serial Reaction Time (ASRT) task (Howard and Howard, 1997), which enables us to separate general skill learning and sequence specific learning. General skill learning refers to the increase in speed as the result of practice and it is relatively independent from sequence structure, while sequence-specific learning refers to the acquisition of sequence-specific knowledge, which results in relatively faster responses for events that can be predicted from the sequence structure vs. those that cannot. Most research, including the Negash et al.'s (2007b) study cited above, has not distinguished these because the tasks used make it difficult to do so. In classical SRT tasks used also by Negash et al. (2007b), the structure of a sequence is deterministic, with the stimuli following a simple repeating pattern as in the series 213412431423, where numbers refer to distinct events. In contrast, in the ASRT task (Howard and Howard, 1997; Remillard, 2008), repeating events alternate with random elements. This means that the location of every second stimulus on the screen is determined randomly. If, for instance, the sequence is 1234, where the numbers represent locations on the screen, in ASRT the sequence of stimuli will be 1r2r3r4r, with r representing a random element. The sequence is thus ‘better hidden’ than in the deterministic SRT task and it is also possible to track sequence-specific learning continuously by comparing responses to the random and sequence elements. This structure is called probabilistic second-order dependency (Remillard, 2008) because to predict element ‘n’ we need only to know element n-2, regardless of element n-1. In this way, the representations of the probabilistic sequences are more abstract and the acquisition of the sequences is also a statistical learning process. One of the outstanding questions in the literature of implicit learning is if there are functional differences in how implicit learning develops in motor vs. cognitive tasks (Foerde et al., 2008; Ashby et al., 2010). The fact that probabilistic sequences with their statistical properties are more ambiguous due to certain transitions being dictated by a context defined by remote events (Remillard, 2008) suggests that learning these sequences might result in more abstract representations than in deterministic sequence learning tasks (for another view see Keele et al., 2003). Moreover, several studies showed that probabilistic sequence learning is related not only to motor, but also to perceptual processes (Song et al., 2008; Nemeth et al., 2009; Hallgató et al., 2013). Based on these considerations, probabilistic sequence-specific learning is presumed to be related relatively more to cognitive skills, while general skill learning is presumed to be related relatively more to motor skills in this specific design. It is a particularly interesting issue how MCI affects the performance on these two aspects of learning.

In the development and stabilization of memory representation for sequences, the processes of consolidation and reconsolidation, are particularly important (Walker et al., 2003; Rickard et al., 2008; Tucker et al., 2011). During the acquisition of sequences we are learning and recalling and reactivating the sequence elements continuously. Recalling or reactivating a previously consolidated memory makes it once again fragile and susceptible to interference, therefore requiring periods of reconsolidation (Walker et al., 2003). These circle processes make possible the continued refinement and reshaping of previously learned motor or cognitive skills in the context of ongoing experience. In experimental designs (fingertapping or SRT tasks) and partly in real-life situations, we are learning sequences arranged in blocks which are separated by shorter or longer time periods. In the beginning of the blocks we reactivate the already consolidated memory traces. Rickard et al. (2008) and Brawn et al. (2010) showed that the separate analysis of the different parts of the learning blocks is crucial in understanding the consolidation of sequence learning. For example, if we analyze only the first part of each of the learning blocks, we can find greater sequence learning effects by controlling the reactive inhibition [i.e., the inhibiting effect of fatigue on learning (Rickard et al., 2008)]. These effects can be particularly relevant in a cognitive impaired population such as MCI. It is important to highlight, however, that Rickard et al. (2008) and Brawn et al. (2010) used explicit and not implicit sequence learning. Thus, the question can be raised whether the pattern of results is the same for implicit learning. We hypothesize dissociation between explicit and implicit sequence learning because several factors, such as fatigue and attentional resources, affect the two types of learning differently (Nissen and Bullemer, 1987; Squire and Zola, 1996; Janacsek and Nemeth, 2013).

It is also a relevant issue that sequence learning does not occur only during practice—online periods—but also between practice periods—during offline periods. The process that occurs during the offline periods is referred to as consolidation and is typically revealed either by increased resistance to interference and/or by improvement in performance, following an offline period (Krakauer and Shadmehr, 2006). The nucleus caudate and ventricle putamen, which are part of the fronto-striato-cerebellar network, play important roles in sequence consolidation (Doyon et al., 2002, 2009b; Doyon and Benali, 2005; Lehericy et al., 2005; Albouy et al., 2008; Debarnot et al., 2009). More recent studies also emphasize the role of the hippocampus in the consolidation of sequence knowledge: for example, Albouy and colleagues (2008) found hippocampus activity using a 24-h delay interval between the learning and testing session. MCI is an ideal avenue to solve the puzzle of sequence consolidation because of the above mentioned neurocognitive background of this cognitive impairment. Although there are several studies focusing on the consolidation of explicit processes in MCI (e.g., Westerberg et al., 2012), to our knowledge no study has investigated the effect of a 24-h offline period on implicit sequence learning in this population so far.

In this study, we investigated sequence-specific and general skill learning in individuals with MCI. In this way we could indirectly investigate the role of the hippocampus and related MTL structures in this learning mechanism. A probabilistic sequence learning task was set up in a prolonged way in order to map the development and consolidation of memories for sequences. We had two main questions here: (1) to which extent can the individuals with MCI learn raw probabilities implicitly, (2) how within-block effects contribute to sequence learning performance. For the second question we hypothesized that the beginning of the learning blocks reflects the processes in which we are picking up high and low frequency triplets and reactivating/recalling the sequence information learned in the previous blocks. As reactivation/recall processes are shown to be related to the hippocampus and related structures (e.g., Gelbard-Sagiv et al., 2008; Xue et al., 2010), we expected weaker learning performance in MCI based on the first half of the blocks compared to the second half of the blocks.

## Materials and methods

### Participants

Seventeen MCI patients and 17 healthy elderly controls participated in the experiment. Diagnoses of MCI were established via a consensus meeting of at least two clinical neurologists and a neuropsychologist using various examinations and tests (e.g., basic laboratory tests, brain MRI, clinical evaluation, Mini Mental State Examination—MMSE). Controls were individuals who: (1) were independently functioning community dwellers, (2) did not have active neurological or psychiatric conditions, (3) had no cognitive complaints, (4) demonstrated a normal neurological behavior, (5) were not taking any psychoactive medications (Negash et al., 2007b).

The MCI and the control group were matched on age (*M*_MCI_ = 61.82, *SD*_MCI_ = 7.70; *M*_control_ = 57.82, *SD*_control_ = 8.47), years of education (*M*_MCI_ = 13.35, *SD*_MCI_ = 2.21; *M*_control_ = 14.18, *SD*_control_ = 2.38) and gender (14 and 15 females, respectively). The groups differed in performance on the MMSE [*t*_(32)_ = −6.31, *p* < 0.001]: the mean score was 26.91 (*SD* = 1.69, range 25–28) for the MCI group and 29.69 (*SD* = 0.48, range 29–30) for the controls. All participants provided signed informed consent agreements and received no financial compensation for their participation. The examinations were conducted at the neuropsychiatric office of the Aladár Petz County Research Hospital.

### Procedure

The ASRT task was administered in two sessions separated by a 24-h interval. Participants were informed that the main aim of the study was to find out just how extended practice affected performance on a simple reaction time task. Therefore, we emphasized performing the task as fast and as accurate as they could. They were not given any information about the regularity that was embedded in the task.

In the first session the ASRT consisted of 20 blocks. As one block took about 1.5–2 min, the first session took approximately 30–40 min. Between blocks, participants received feedback on the screen about their overall reaction time and accuracy, then had a rest of between 10 and 20 s before starting a new block. Session 2 lasted approximately 22–30 minutes, as the ASRT consisted of 15 blocks.

The computer program selected a different ASRT sequence for each participant based on a permutation rule, such that each of the six unique permutations of the four possible stimuli occurred. Consequently, six different sequences were used across participants while the sequence within participants was identical during Session 1 and Session 2 (Howard and Howard, 1997; Nemeth et al., 2010).

### The alternating serial reaction time (ASRT) task

Sequence learning was measured by the “Catch the dog” version (Nemeth et al., 2010) of the ASRT task (Howard and Howard, 1997). In this ASRT task, a stimulus (a dog's head) appears in one of four empty circles on the screen and participants have to press the corresponding button when it occurs. The computer is equipped with a special keyboard with four heightened keys (Y, C, B, and M on a Hungarian keyboard; equivalent to Z, C, B, M on a US keyboard), each corresponding to the circles in a horizontal arrangement.

Unbeknownst to participants, the appearance of stimuli follows a predetermined order. As stimuli are presented in blocks of 85 stimuli, the first five button pressings are random for practice purposes, then an 8-element alternating sequence (e.g., 2r3r1r4r, where numbers represents the four circles on the screen and r represents random elements) repeats ten times. Because of this structure, some triplets or runs of three consecutive events occur more frequently than others. For example, in the above illustration, 1_4, 2_3, 3_1, and 4_2 (where “_” indicates the middle element of the triplet) would occur often because the third element (bold numbers) could be derived from the sequence or could also be a random element. In contrast, 1_3 or 4_1 would occur less frequently because in this case the third element could only be random. Following previous studies, we refer to the former as high-frequency triplets and the latter as low-frequency triplets. Note that the final event of high-frequency triplets is therefore more predictable from the initial event when compared to the low-frequency triplets [also known as non-adjacent second-order dependency (Remillard, 2008)]. Therefore, for each stimulus we determined whether it was the last element of a high- or low-frequency triplet.

There are 64 possible triplets (4^3^, 4 stimuli combined for three consecutive events) in the task. Out of these triplets, 16 are high frequency triplets, each of them occurring on approximately 4% of the trials, about five times more often than the low-frequency triplets. Thus, approximately 64% of all trials are high-frequency triplets and the remaining 36% of trials are low-frequency ones.

Previous studies have shown that as people practice the ASRT task, they come to respond more quickly to the high- than low-frequency triplets, revealing sequence-specific learning (Howard and Howard, 1997; Howard et al., 2004; Song et al., 2007). In addition, general skill learning is revealed in the ASRT task in the overall speed with which people respond, regardless of the triplet types. Thus, we are able to obtain measures of both sequence-specific and general skill learning in the ASRT task.

### Statistical analyses

To facilitate data processing, the blocks of ASRT were organized into epochs of five blocks. The first epoch contains blocks 1–5, the second blocks 6–10, etc. (Bennett et al., 2007; Barnes et al., 2008). As participants' accuracy remained very high (98.1% for the MCI and 99.2% for the control group) throughout the test (similarly to previous studies, e.g., Howard and Howard, 1997; Nemeth et al., 2010), we focused on reaction time (RT) for the analyses reported. For RTs, we calculated medians for correct responses only, separately for high and low frequency triplets and for each participant and each epoch.

To compare the overall learning between the groups, RTs were analyzed by a mixed design ANOVA on the 7 epochs of Session 1 and 2 with TRIPLET (2: high vs. low) and EPOCH (1–7) as within-subjects factors and GROUP (MCI vs. control) as a between-subjects factor. For exploration of offline changes in the 24-h delay period, a similar ANOVA was conducted including only the last epoch of Session 1 and the first epoch of Session 2. All significant results are reported together with the η^2^_*p*_ effect size and Greenhouse–Geisser ε correction factors where applicable. Planned comparisons and *post-hoc* analyses were conducted by Fisher's LSD pairwise comparisons.

## Results

### Do the MCI and the control group differ in overall sequence learning?

**Figure 1:**
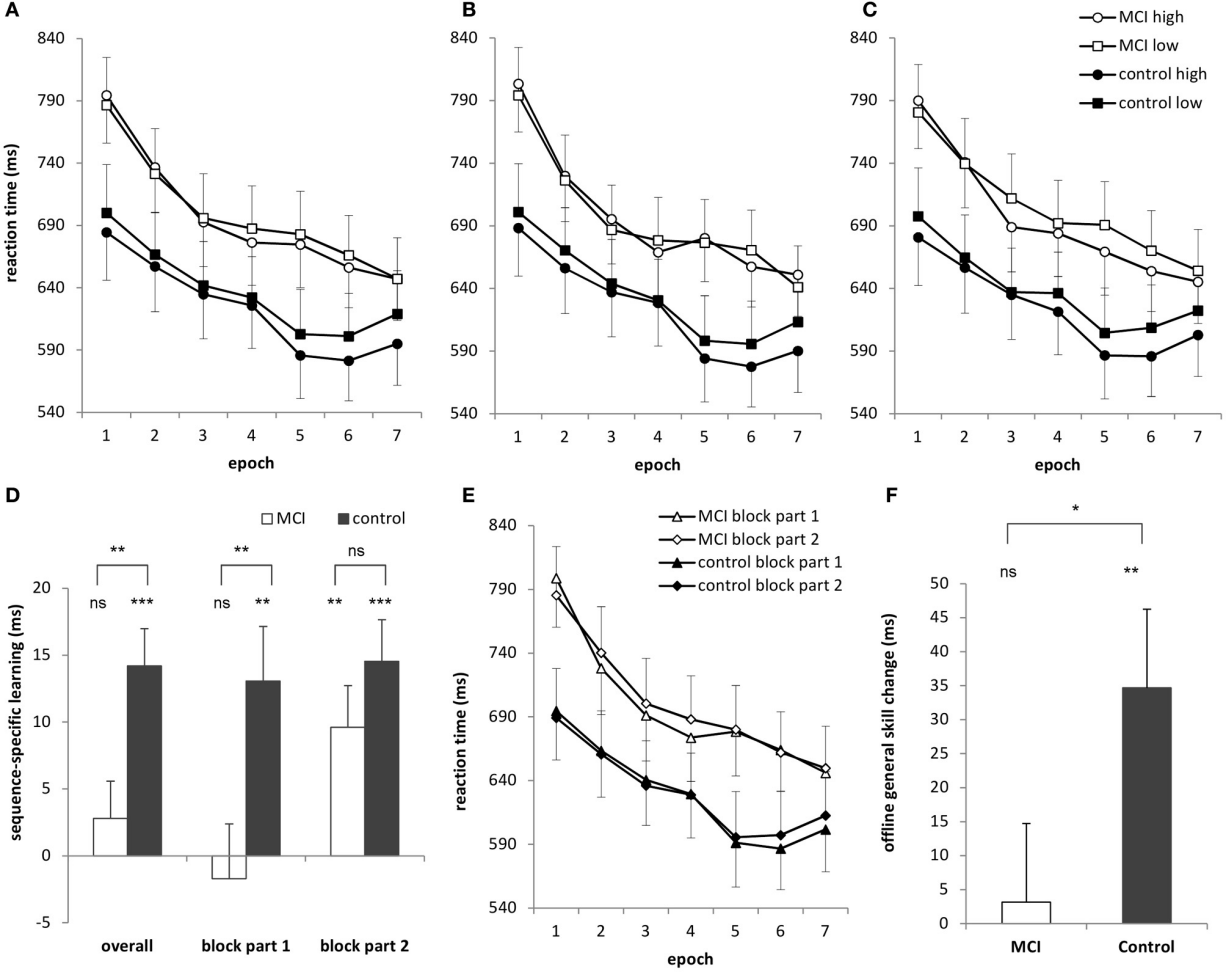
**(A)** Sequence learning across 7 epochs (35 blocks) for the MCI and control group. Circles represent RTs for high-frequency triplets and squares represent RTs for low-frequency triplets. **(B)** Learning curves for the first part of each block and **(C)** the second part of each block. **(D)** Sequence-specific learning (measured by the RTs for the low- minus high-frequency triplets) for the MCI and control group is plotted for the overall, first block-part and second block-part learning measures. Overall, the MCI group did not show significant sequence-specific learning, which was caused mainly by the learning performance in the first part of the blocks. The learning performance in the second part of the blocks was similar in the groups. **(E)** General reaction times are plotted for the first and second parts of the blocks for the MCI and control group, separately. The MCI group was slower in the second parts of the blocks compared to the first parts of the blocks, but only in Session 1. The control group showed a similar pattern, but in Session 2. **(F)** Offline general skill changes (measured as the RT difference between Epoch 4 and Epoch 5, irrespectively of the triplet types) over the 24-h delay are plotted for the MCI and the control group with significant offline improvement for the controls only. Error bars represent standard error of mean. ns, non-significant, ^*^p < 0.05, ^**^p < 0.01, ^***^p < 0.001.

The ANOVA revealed significant *sequence-specific learning* [indicated by the significant main effect of TRIPLET: *F*_(1, 32)_ = 18.50, η^2^_*p*_ = 0.37, *p* < 0.001] such that RTs were faster on high than on low frequency triplets (Figure 1A). The groups differed in the extent of this sequence-specific learning [shown by the significant TRIPLET × GROUP interaction: *F*_(1, 32)_ = 8.31, η^2^_*p*_ = 0.21, *p* = 0.007]: the MCI group was 2.80 ms faster on high than on low frequency triplets (*p* = 0.32) while this difference was 14.20 ms for the controls (*p* = 0.001). Thus, only the controls acquired the sequence-specific knowledge overall.

The ANOVA also revealed *general skill learning* [shown by the significant main effect of EPOCH: *F*_(6, 192)_ = 42.70, η^2^_*p*_ = 0.57, *p* < 0.001], such that RTs decreased across epochs, irrespective of the triplet type. This decrease was slightly different for the groups [EPOCH × GROUP interaction: *F*_(6, 192)_ = 2.33, η^2^_*p*_ = 0.07, *p* = 0.078]: RTs decreased steeper in the MCI group (153 ms from the first epoch to the last epoch) than in the controls (95 ms). This difference was mainly caused by the MCI group's relatively slower RTs in the first epoch compared to that of the controls (790 vs. 692 ms, *p* = 0.07). This difference diminished for the last epoch (647 vs. 607 ms, *p* = 0.41). Other interactions were not significant (*p*s > 0.17).

Although the MCI and the control group performed with similar RTs [main effect of GROUP: *F*_(1, 32)_ = 1.99, *p* = 0.17], we re-ran our analyses using z-transformed RTs to confirm our findings. The ANOVA revealed sequence-specific learning [significant main effect of TRIPLET: *F*_(1, 32)_ = 43.77, *p* < 0.001] with significantly smaller learning for the MCI than for the control group [TRIPLET × GROUP interaction: *F*_(1, 32)_ = 4.01, *p* = 0.05]. After the z-transformation, the EPOCH × GROUP interaction was not significant [*F*_(6, 192)_ = 1.26, *p* = 0.31], suggesting a similar level of general skill learning in the two groups.

### Is there any within-block effect on learning? are these effects different in the MCI and the control group?

A fine-grained analysis of the data can give us a deeper insight into the mechanisms of the development of sequence representation; therefore, it can also help to better understand the above reported sequence-learning deficit in MCI compared to controls. Analyzing the learning data by splitting each block into two halves is an excellent approach for exploring these questions. Therefore, we conducted a mixed design ANOVA on the data shown in Figures 1B,C with TRIPLET (2: high vs. low frequency), EPOCH (7: 1–7) and PART (2: first vs. second half of blocks) as within-subject factors and GROUP (2: MCI vs. control) as a between-subject factor.

**Figure 2:**
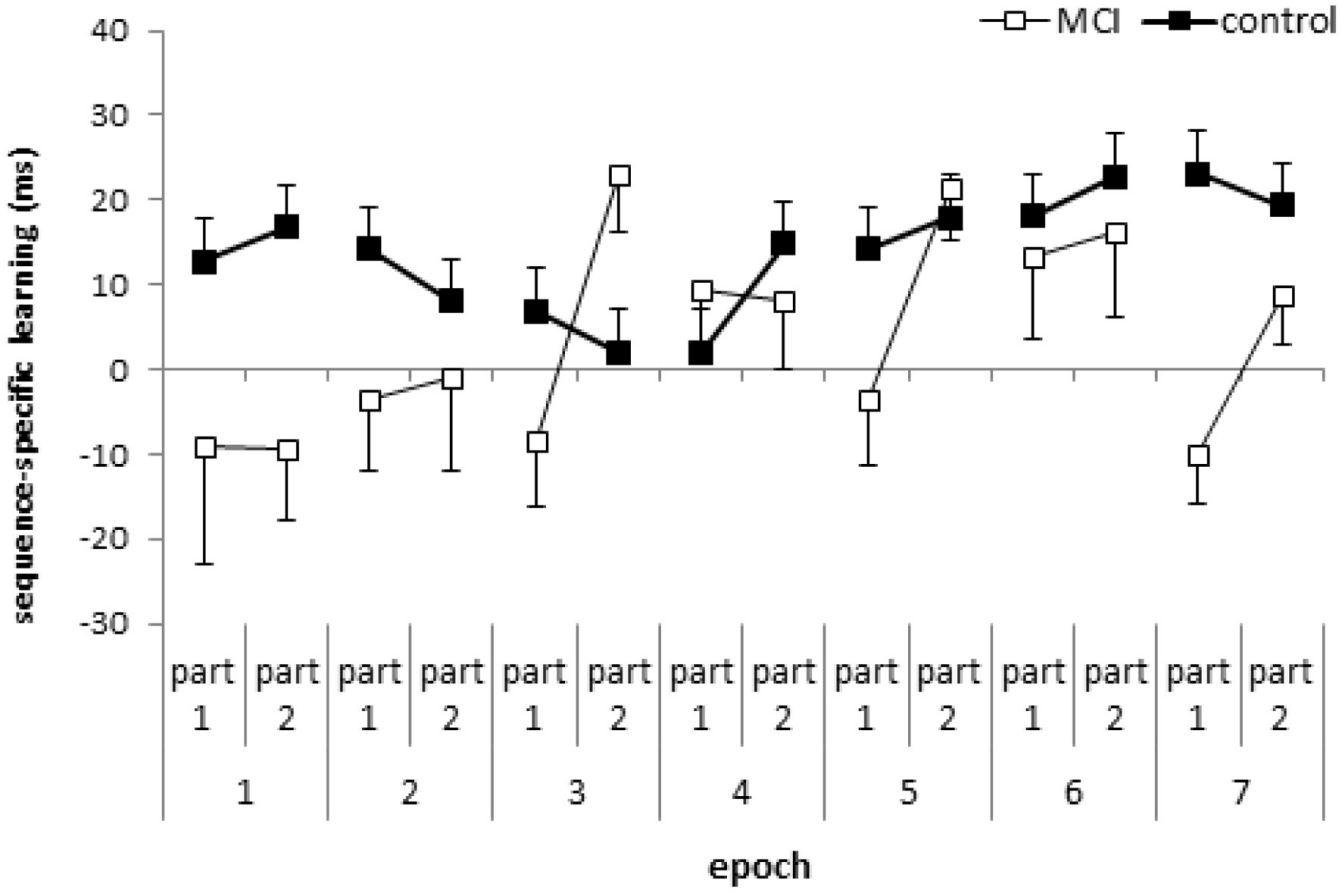
**Sequence-specific learning (measured by the RTs for the low- minus high-frequency triplets) in the first and second parts of the blocks, collapsed into epochs, is plotted for the MCI and control group.** Error bars indicate standard error of mean.

The ANOVA revealed significant sequence-specific learning overall [main effect of TRIPLET: *F*_(1, 32)_ = 18.27, η^2^_*p*_ = 0.36, *p* < 0.001] with smaller learning for the MCI group compared to controls [4 vs. 14 ms; TRIPLET × GROUP interaction: *F*_(1, 32)_ = 5.62, η^2^_*p*_ = 0.15, *p* = 0.02; Figure 1D]. Interestingly, taking the PART of the blocks into account, we found a significant TRIPLET × PART interaction [*F*_(1, 32)_ = 4.43, η^2^_*p*_ = 0.12, *p* = 0.04]: the sequence-specific learning was greater in the second part of the blocks compared to the first part (6 vs. 12 ms). Although the TRIPLET × PART × GROUP interaction did not reach significance [*F*_(1, 32)_ = 2.62, η^2^_*p*_ = 0.08, *p* = 0.12], planned comparisons revealed that the controls showed a similar extent of sequence-specific learning in the first and the second part of the blocks (13 and 14.5 ms, *p* = 0.73). In contrast, the MCI group showed higher sequence-specific learning in the second part of blocks than in the first part (1.7 vs. 9.6 ms, *p* = 0.01). All of these learning measures were significant (*p*s < 0.004), except for the first part of the blocks in the MCI group (*p* = 0.68). Thus, the group difference in sequence learning that we found in the previous analysis was driven mainly by the first part of the blocks (Figure 2), where the extent of sequence-specific learning was different between groups (*p* = 0.01), while they were similar in the second part of the blocks (*p* = 0.22).

The ANOVA computed on z-transformed data confirmed our findings, as the TRIPLET × PART × GROUP interaction was significant [*F*_(1, 32)_ = 5.93, *p* = 0.02]. The MCI group showed significant sequence-specific learning only in the second halves of the blocks (*p* < 0.001) but not in the first halves (*p* = 0.29). In contrast, the controls exhibited significant sequence-specific learning both in the first and second parts of the blocks (*p*s < 0.001).

In the case of general skills, the ANOVA showed a significant improvement across epochs [main effect of EPOCH: *F*_(6, 192)_ = 42.42, η^2^_*p*_ = 0.57, *p* < 0.001], with a trend toward group differences [EPOCH × GROUP interaction: *F*_(6, 192)_ = 2.46, η^2^_*p*_ = 0.07, *p* = 0.06]. This latter effect is similar to the results of the previous analysis finding that RTs decreased steeper in the MCI group (149 ms from the first epoch to the last epoch) than in the controls (87 ms). This difference, however, diminished when analyzing z-transformed data: [EPOCH × GROUP interaction: *F*_(6, 192)_ = 1.23, *p* = 0.32].

There was also a trend for different degrees of general skill improvement in the first and second part of the blocks [EPOCH × PART interaction: *F*_(6, 192)_ = 1.91, η^2^_*p*_ = 0.06, *p* = 0.08]: the speed-up from the first to the last epoch was 123 ms when analyzing only the first parts of the blocks, while it was slightly smaller in the case of the second parts of the blocks (106 ms). This was caused mainly by being faster in the second half of the blocks at the beginning of the task (737 vs. 746 ms in the first epoch), with a reverse pattern for the end of the task (631 vs. 624 ms in the last epoch). This effect remained and even became stronger after z-transforming the RTs [EPOCH × PART interaction: *F*_(6, 192)_ = 6.80, *p* < 0.001].

Groups further detailed this picture [significant EPOCH × PART × GROUP interaction: *F*_(6, 192)_ = 2.22, η^2^_*p*_ = 0.07, *p* = 0.04; Figure 1E] as the MCI group was 12 ms faster in the first parts of the blocks compared to the second parts in Session 1 (*p* = 0.004) but showed similar RTs in Session 2 (1 ms difference between the RTs of the first and second parts of the blocks, *p* = 0.73). In contrast, the control group performed the task with similar RTs in Session 1 (2.6 ms difference, *p* = 0.51) but was 8.6 ms faster at the beginning of the blocks compared to the second parts in Session 2 (*p* = 0.01). This difference, however, disappeared when using z-transformed data [EPOCH × PART × GROUP interaction: *F*_(6, 192)_ = 0.02, *p* = 0.33]. No other main effects of interactions were significant (*p*s > 0.21).

### Is there any change in learning in the 24-h delay?

For the exploration of the offline changes in the 24-h delay period, ANOVA was conducted with TRIPLET (2: high vs. low frequency) and EPOCH (2: the last epoch of Session 1 and the first epoch of Session 2) as within-subject factors and GROUP (2: MCI vs. control) as a between-subject factor.

The ANOVA revealed sequence-specific learning [indicated by the significant main effect of TRIPLET: *F*_(1, 32)_ = 19.68, η^2^_*p*_ = 0.38, *p* < 0.001] which was retained across the sessions [TRIPLET × EPOCH interaction: *F*_(1, 32)_ = 0.51, η^2^_*p*_ = 0.02, *p* = 0.48]. The groups did not differ either in overall sequence-specific knowledge [TRIPLET × GROUP interaction: *F*_(1, 32)_ = 0.19, η^2^_*p*_ = 0.01, *p* = 0.67] or in the offline change of this knowledge between the sessions [TRIPLET × EPOCH × GROUP: *F*_(1, 32)_ = 1.63, η^2^_*p*_ = 0.05, *p* = 0.21].

In contrast, there was an offline improvement in general skills [main effect of EPOCH: *F*_(1, 32)_ = 5.32, η^2^_*p*_ = 0.14, *p* = 0.028], with faster RTs in the first epoch of Session 2 compared to the last epoch of Session 1 (Figure 1F). This change was slightly different between groups [EPOCH × GROUP interaction: *F*_(1, 32)_ = 3.69, η^2^_*p*_ = 0.10, *p* = 0.064]: the MCI group showed no between-session speed-up (3 ms, *p* = 0.79) while the controls did (34.7 ms, *p* = 0.005). The ANOVA on z-transformed RTs confirmed this result, showing a weaker consolidation of general skills for the MCI than for the control group [marginally significant EPOCH × GROUP interaction: *F*_(1, 32)_ = 3.85, *p* = 0.06]. Other interactions involving the GROUP were not significant (*p*s > 0.71).

We also conducted a consolidation analysis taking the first and second parts of the blocks into account and found similar results, with significant group differences in offline general skill changes [EPOCH × GROUP interaction: *F*_(1, 32)_ = 4.30, η^2^_*p*_ = 0.12, *p* = 0.046]. The offline change in general skills was significant for the control group (35.8 ms faster at the beginning of Session 2 compared to the end of Session 1, *p* = 0.004) but not significant for the MCI group (1.65 ms difference, *p* = 0.89).

## Discussion

Our goal was to investigate the acquisition of sequence knowledge in Mild Cognitive Impairment. We used a task that allows differentiating between sequence-specific and general skill learning. At first, based on the standard ASRT analysis we found that individuals with MCI showed weaker implicit probabilistic sequence learning than the healthy aged group. However, once we dug deeper and considered only the second half of each learning block, we found similar learning performances in the MCI as in the healthy aged group. Thus, the overall sequence-specific learning in MCI depends on which part of each learning block is considered. In the case of general reaction time, the MCI group was faster in the first part of the blocks compared to the second part in Session 1. The healthy aged group showed a similar pattern, except in Session 2. We were able to demonstrate that general skill consolidation over a 24-h delay period was different in MCI and in the healthy aged group. The latter group showed offline improvement in general reaction time while the MCI group did not show this speed-up effect. We believe our study to be the first one that uses an implicit sequence learning task with second-order dependency in individuals with MCI.

Our results partly contradict but partly support the findings of Negash and his colleagues (2007b), who showed learning with a deterministic SRT task in MCI but not in the Contextual Cueing task (Chun and Jiang, 1998). The impaired sequence learning that we found in MCI could be due to the more difficult and more complex sequence structure in our task, compared to the one used by Negash et al. (2007b). Another possibility is that deterministic and probabilistic sequence learning tasks are qualitatively different: the latter with their statistical properties are more ambiguous due to higher order associations in which a current event is predicted not by the preceding event but by the context of more remote events (Cohen et al., 1990; Keele et al., 2003). Thus, our result of impaired sequence learning in MCI is more similar to the results of the Contextual Cueing task in Negash et al.'s study. The Contextual Cueing task relies on visual search (e.g., find a horizontal T on the screen), which is generated within a background of some repeated distractor configuration (unknown to participants) providing a contextual cue to the location of the target. As a result of practice, the participants detect the target-stimulus in repeated configurations faster than in random configurations, even though they are not aware of the repeated distractors. This task calls on different neural systems than the SRT task (MTL-hippocampus vs. the frontal-striatal-cerebellar system; Curran, 1998; Honda et al., 1998; Chun and Jiang, 1999; Gomez-Beldarrain et al., 1999; Manns and Squire, 2001; Willingham et al., 2002). Despite these differences in the involvement of different neural systems, our results suggest that the MTL and the hippocampal formation are also somehow involved in probabilistic sequence learning measured by the ASRT task. The within-block analysis can help us specify the nature of this involvement.

The result that the overall sequence-specific learning depends on whether we consider the first part or the second part of each learning block supports the suggestion of Rickard et al. (2008), who stressed the importance of the within-block position effect. However, we did not find a fatigue effect within the block in either group. Moreover, in the MCI group we showed significant overall sequence-specific learning when only taking the second part of the learning blocks into account, suggesting a warm-up or priming effect (cf. Figure 2). The fact that the MCI group exhibited significant sequence-specific learning in the second part of the blocks but not in the first part, suggests that the processes are qualitatively different between the first and the second part of the learning blocks. In the beginning of the blocks we have to recall and reactivate the sequence structure partly learned already in the previous blocks. The second part of each block might be responsible for the utilization and/or proceduralization of the sequence knowledge. Based on these assumptions, we claim that the detection of probabilities in the reactivation/recall phase is somehow impaired in MCI. In addition, as MTL structures, including the hippocampus are primarily affected in MCI (Jack et al., 1997; Dickerson and Sperling, 2008) and we found impaired sequence learning in the first part of learning blocks, the reactivation/recall of the sequence knowledge in the beginning of the blocks might be more MTL-dependent than in the second part. However, more studies are needed to confirm this suggestion.

These within-block effects also open a window to the similarities and dissimilarities between learning performance on the ASRT and the Contextual Cueing task. Although several neuropsychological studies have showed dissociation on the performance of these tasks, showing evidence of the different neurocognitive background (Howard et al., 2006; Negash et al., 2007a; Barnes et al., 2010; Simon et al., 2011), our results suggest that these two tasks somehow involve similar processes but only in the first part of the ASRT blocks. In this part of the blocks the reactivation/recall of the previously learned regularities is prominent. Moreover, in order to recover the previously acquired sequence memories, picking up the context information of the items at the beginning of each block is essential. As previous studies showed, these processes are linked to the hippocampus and related MTL structures (Wood et al., 2000; Gelbard-Sagiv et al., 2008; Xue et al., 2010). In sum, learning performance in specific parts of the ASRT seems to rely on the involvement of the hippocampus and related MTL structures.

Regarding general reaction times, we found that in Session 1 the MCI group was faster in the first part of the learning blocks compared to the second part, while this pattern was present for the control group in Session 2. Generally, slower RTs at the end of learning blocks than at the beginning suggest a build-up of fatigue within each block. This fatigue effect emerges later for the controls than for the MCI group. These results partly support the findings of Rickard and his colleagues (2008), who showed this fatigue effect masking some aspects of learning performance in a fingertapping task. Since the MCI group showed significant sequence-specific learning in the second half of the blocks, in spite of the fact that they were generally slower due to fatigue, we can claim that the impaired sequence-specific learning in the MCI group is not caused by this fatigue effect in our study.

Previous studies argue that the caudo-ventral putamen (Doyon and Benali, 2005; Debarnot et al., 2009) and the hippocampus (Albouy et al., 2008) can both play a role in the consolidation of sequence learning. Since MTL structures, including the hippocampus, are mostly affected by MCI (Dickerson and Sperling, 2008), our results that the MCI group did not forget the sequence in the 24-h delay period might suggest that these structures are not essential for the consolidation of sequence-specific knowledge, though they might affect the consolidation of general skill learning. This latter finding is in line with previous studies using fingertapping tasks (e.g., Walker et al., 2003), suggesting that general skill learning in our design might share similar neurocognitive background with motor learning. However, future studies need to clarify these similarities.

In sum, our findings that the detection of probabilities in the reactivation/recall phases of the learning is impaired in MCI draw attention to the importance of the hippocampus and the related MTL structures in the development of sequence memory representation. Our results add detail to the picture regarding background processes of sequence acquisition and consolidation and refine Negash et al.'s (2007b) final conclusion that adapting to environment is preserved in MCI. Based on our findings, we believe that the reactivation phase of the detection of probabilities is impaired in MCI. If further studies with different methods, including functional brain mapping, confirm this view, it could lead to the development of more focused and more effective prevention and rehabilitation programs for minor and major cognitive disorders.

### Conflict of interest statement

The authors declare that the research was conducted in the absence of any commercial or financial relationships that could be construed as a potential conflict of interest.
